# Dietary effects of protected fat, glycerol, and soybean meal on performance, physiological parameters, carcass characteristics, and behavioral measurements of late-fattening Hanwoo steers under heat stress conditions

**DOI:** 10.5713/ab.23.0503

**Published:** 2024-04-25

**Authors:** Jun Sik Woo, Gyeong Rim Ryu, Jeong Hoon Kim, Sun Sik Jang, Hong Gu Lee, Keun Kyu Park

**Affiliations:** 1 Department of Animal Science and Technology, Konkuk University, Seoul 05029, Korea; 2 National Institute of Animal Science, Rural Development Administration, Cheonan 31000, Korea; 3 Cargill Agri Purina Inc., Seongnam 13630, Korea; 4 National Institute of Animal Science, Rural Development Administration, Pyeongchang 25340, Korea

**Keywords:** Glycerol, Hanwoo Steers, Heat Stress, Late-fattening Period, Protected Fat, Soybean Meal

## Abstract

**Objective:**

This study aimed to determine the effects of increasing energy and protein levels in diets by including protected fat (PF), glycerol (GL), and soybean meal (SBM) on growth performance, physiological parameters, carcass characteristics, and behavioral measurements of late-fattening Hanwoo steers under heat stress conditions.

**Methods:**

Thirty-six steers (initial body weight, 724.9±58.3 kg; age, 25.5±0.4 month) were assigned into control (total digestible nutrient [TDN] 76%, crude protein [CP] 15%), PF (TDN 83.6%, CP 15%), PF+GL (TDN 83.6%, CP 15%) and PF+GL+SBM (TDN 83.6%, CP 16.5%) by randomized complete block design for a total of 16 weeks with division of 4-week periods. The average temperature-humidity index was 87.0 (1st period; severe), 82.8 (2nd; moderate), 71.4 (3rd; comfort), and 68.1 (4th; comfort).

**Results:**

The dry matter intake (DMI) showed no treatments differences during the whole experiment. However, DMI in 1st and 2nd period decreased by approximately 30% and 10% compared to 4th period, respectively. Higher average daily gain and feed conversion ratio were noted for treatments compared to control at both 1st and 2nd period (p<0.05). There were no treatment effects on rectal temperature (RT), cortisol, and behaviors during the entire experiment. However, both RT and cortisol in 0, 1st and 2nd period were higher than those of 3rd and 4th period (p<0.05). Carcass yield and grade remained unaffected by increasing TDN and CP levels. Behavioral changes in the hot season (1st period) included reduced lying (43%), increased standing (48%), decreased walking (62%), and decreased eating (38%) (p<0.05), with an increase in drinking by 54%. Rumination during standing was 53% higher, while rumination during lying was about 33% lower compared to the post-hot season (3rd period) (p<0.05).

**Conclusion:**

Dietary supplementation of protected fat in late-fattening Hanwoo steers under heat stress had a positive effect on preventing a reduction in performance.

## INTRODUCTION

Due to global warming, the mean global surface temperature has increased by 1.1°C during the last decade (2011 through 2020) compared to 1850 through 1990 and is predicted to reach 1.5°C in the near future (2021 through 2040) [1]. Heat stress in ruminants due to extreme weather conditions can negatively affect dry matter intake (DMI), growth rate, gain to feed ratio, overall health, milk yield or fertility [2,3]. The stress can reduce rumen activity and motility, which can slow down the fractional passage rate of digesta through the gastrointestinal tract [4]. This reduction in rumen activity primarily results in a decrease in DMI and total volatile fatty acid (VFA) production in the rumen [5]. Consequently, effective methods of supplemental energy supply should be considered to prevent a productivity decrease due to energy deficiency under heat stress conditions.

Ruminants exposed to heat stress have increased maintenance energy requirements because of an increasing amount of energy needed to maintain body temperature and homeostasis for regulating metabolic physiological activities. On the other hand, because feed intake decreases to reduce body heat production, the increased energy requirement cannot be met. It leads to a decrease in productivity along with a decrease in body weight (BW) gain [6].

Several studies have attempted to enhance dietary energy by introducing rumen-protected fat or glycerol [7–10]. Glycerol is rapidly fermented by microorganisms in the rumen and mostly converted to propionate. Feeding glycerol results in decreased acetate and acetate to propionate ratio but no effect on butyrate and total VFA [10]. Propionate is rapidly absorbed into rumen walls with high conversion efficiency to glucose [9]. Therefore, glycerol has been established as a substitute for corn and is absorbed by the body to support glucose synthesis and lipogenesis [8]. Protected fat is mainly utilized to increase the energy density of feeds by providing more fat to the duodenum without interfering fermentation of rumen microorganisms [11]. However, particularly in beef cattle, the data of feeding protected fat on productivity are very limited. Moreover, little is known about the effects of increasing energy and protein levels in the diets of heat-stressed Hanwoo steers during late-fattening period on growth performance and blood parameters. Therefore, this study was conducted to determine the effects of increasing energy and protein levels by using protected fat, protected fat plus glycerol, and soybean meal on performance, physiological parameters, carcass characteristics, and behavioral measurements of late-fattening Hanwoo steers under heat stress conditions.

## MATERIALS AND METHODS

### Animal care

The experimental protocol was reviewed and approved by the Institutional Animal Care and Use Committee at Konkuk University (Approval number: KU21095).

### Animal and feeding trial

Thirty-six Hanwoo steers (initial BW, 724.9±58.3 kg; age, 25.5±0.4 month) were allocated into four treatments by randomized complete block design according to BW to 12 pens (3 steers/pen). Based on National Institute Animal Science of Korea (NIAS) [12], the treatment group increased the total digestible nutrient (TDN) and crude protein (CP) levels by an additional 10% compared to control: control (no supplements; TDN 76%, CP 15%), PF (protected fat; TDN 83.6%, CP 15%), PF+GL (protected fat + glycerol; TDN 83.6%, CP 15%) and PF+GL+SBM (protected fat+ glycerol + soybean meal; TDN 83.6%, CP 16.5%). The TDN was calculated using 0.93×CP+0.92×(1+ether extract [EE]–ash–CP–neutral detergent-insoluble fiber [NDF])+ 0.75×(NDF–acid detergent-insoluble lignin [ADL])×(1–ADL^2/3^/NDF^2/3^) suggested by Conrad et al [13].

Glycerol was 99.5% purified glycerol (LG Household & Health Care Co., Ltd., Seoul, Korea). Protected fat (Energy booster 100; Milk Specialties Global, Eden Prairie, MN, USA) was a type of prilled fat based on palm oil consisting of 99.5% free fatty acids, of which myristic acid (C 12:0) 2.5%, palmitic acid (C 16:0) 28.0%, stearic acid (C 18:0) 45.0%, oleic acid (C 18:1) 8.3%, linoleic acid (C 18:2) 1.5%, and linolenic acid (C 18:3) 0.1%. The experimental diet was prepared as a total mixed ration (TMR) using a horizontal stationary mixer (SI-2A-350S; Silti, Goyang, Korea). The addition of glycerol alone in the diet formulation of late-fattening Hanwoo steers cannot increase energy by 10% due to low energy content of glycerol. Thus, glycerol only was excluded from the treatment groups in this experiment. Thus, the PF+GL treatment was formulated with glycerol and protected fat in a 50:50 ratio based on TDN, and the ingredients and chemical composition of the TMR diets are presented in Tables 1 and 2, respectively.

This experiment was conducted from July 9, 2021 to October 28, 2021 for a total of 112 days (16 weeks) with divisions of 4 week periods. The BW was measured at the beginning of the experiment and every 4 weeks before morning feeding until the end of the experiment. Feeds were offered *ad libitum* twice at 0700 and 1700 h daily and residuals were measured before the next morning feeding. Water and mineral blocks were available without constraint. The feed conversion ratio (FCR) was calculated using DMI (kg)/BW gain (kg).

Animals were transported to a local slaughterhouse (Bucheon, Korea) located one hour away from the experimental site and slaughtered the following day. After stunning with a captured bolt, the animals were slaughtered in a conventional manner. The carcasses after death 24 h were evaluated by the carcass grading system for the Ministry of Agriculture, Food and Rural Affairs (2019) [14].

### Physiological and blood parameters

Rectal temperature (RT) measurements and blood collection were performed at the beginning of the experiment and every 4 weeks throughout the experiment. The RT was measured at 1400 h using a thermometer (KD-133; Polygreen Co., Ltd., Berlin, Germany) after blood collection, the time when heat stress reaches its peak. For accurate RT measurement of animals, the thermometer was inserted at a depth of 3 cm into the rectum and kept in contact with the mucosa for 1 minute [15].

Blood was collected from the jugular venipuncture at 20 mL per steer and stored in non-heparinized vacutainers (10 mL; Becton-Dickinson, Franklin Lakes, NJ, USA) and ethylenediaminetetraacetic acid-treated vacutainers (10 mL; Becton-Dickinson, USA). The collected serum samples were subjected to centrifugation at 2,700 g for 15 minutes at 4°C and transferred to 1.5 mL tubes (Eppendorf AG, Hamburg, Germany) for storage at –80°C until further analysis.

Serum samples were subsequently assayed for glucose, triglyceride (TG), total cholesterol (TCHO), and blood urea nitrogen (BUN) utilizing an automated chemistry analyzer (CHEM 7000i; Fujifilm, Tokyo, Japan). Serum cortisol levels were determined using a bovine ELISA test kit (MBS70325; MyBioSource Inc., San Diego, CA, USA), and analytical reagents for the determination of non-esterified fatty acids (NEFA) were analyzed using WAKO (Osaka, Japan).

### Analysis of chemical composition

All diet samples were subjected to a drying process in a forced-air oven for a duration exceeding 24 hours at 105°C and subsequently ground using a Wiley mill (Model 4; Thomas Scientific, Swedesboro, NJ, USA) through a 2 mm screen. These diet samples were analyzed to determine their composition, including dry matter (DM; method 930.15), CP (method 976.05), EE (method 2003.05), ash (method 942.05), crude fiber (CF; method 962.09), NDF (method 2002.04), acid detergent-insoluble fiber (ADF), and lignin (method 973.18), as following the guidelines of AOAC [16].

### Measurement of ambient temperature and relative humidity

The experiments were conducted in a typical Korean beef farm located at latitude 37.90092507299778 and longitude 126.991961753878. Climatic data were recorded every 10 minutes using four automatic ambient temperature (AT) and relative humidity (RH) loggers (MHT-381SD; Lutron Electronics Inc., Coopersburg, PA, USA) situated in the pen. The recorded data were collected every 4 weeks and the temperature-humidity index (THI) was calculated using the equation ([1.8×T_db_+32]–[0.55–0.0055×RH]×[1.8×T_db_–26]; Tdb, dry bulb temperature [°C]; RH, relative humidity [%]) given by NRC [17]. The minimum, maximum, and average of AT, RH, and THI for each period (4 weeks) are presented in Table 3.

### Observation of animal behaviors

Animal behaviors were observed through a closed-circuit television (CCTV, DS-2CE16D0T-IRP 3.6 mm; HIKVISION, Seoul, Korea), and two steers per treatment were selected and recorded consecutively for 3 days. Two CCTV cameras were installed diagonally in front and behind each pen to allow observation of the entire pen without blind spots.

The observations were divided into seven categories: lying, total standing, walking, eating, drinking, rumination during standing, and rumination during lying (Table 4). Rumination behavior included both lying and standing, and standing included walking, feeding, rumination, and drinking behaviors. The behavioral analysis was conducted based on the heat stress indicators for Hanwoo steers in the fattening period suggested by NIAS (2022) [6] to compare the differences between hot and post-hot seasons by selecting and measuring the days when the stress levels of Severe (THI 85–99; 1st period) and Comfort (THI 68–75; 3rd period) lasted for more than 5 days, respectively.

### Statistical analysis

Statistical analyses of all experimental data were conducted using the MIXED procedure of SAS (SAS Inst. Inc., Cary, NC, USA). The statistical model of performance and carcass data included that dietary treatments were processed as fixed effects, and pens were treated as random effects. The pen including three steers was considered as the experimental unit. Behavioral data included that periods were processed as fixed effects, and animals were treated as random effects. Data of blood profiles and RT were analyzed to a repeated measurement over time using the procedure of Littell et al [18]. Dietary treatments, days, and their interaction were included in the statistical models as fixed effects, and pens were treated as random effects. The compound symmetry was used to ensure that measures always were qualified for the same variance and that all pairs of measures for the same animal were equally correlated. Least squares means was used to calculate the mean values of each treatment and compared using the PDIFF option. Superscript was used when the treatment and period effects were significant. Statistical differences were declared at p<0.05.

## RESULTS AND DISCUSSION

### Animal performance

The mean, minimum, and maximum values of AT, RH, and THI for a total of 16 weeks from the 1st to 4th period of this experiment were all different by periods (p<0.05). The average AT, RH, and THI of the 1st period, which was the highest heat stress period during the whole experiment, were 32.8°C, 74.1%, and 87.0, respectively. These values showed a heat stress condition in the severe (THI 85 to 99) stage according to THI chart of NIAS (2022) [6] for fattening Hanwoo steers. The average values of the 2nd period were 27.5°C, 76.5%, and 82.8, respectively, indicating a moderate (THI 82 to 84) stage. The average AT of the 2nd period was lower than that of the 1st period (p<0.05), but the RH was higher (p<0.05). After the hot season, the average AT, RH, and THI of the 3rd period decreased to 25.1°C, 72.3%, and 71.4, respectively, and, in the 4th period, to 21.3°C, 56.5%, and 68.1, respectively. Both of these periods were regarded under the comfort (THI 75 or less) condition according to THI chart of NIAS (2022) [6] (Table 3).

The BW, DMI, average daily gain (ADG), and FCR of animals measured at initial and every 4 weeks during the experiment are presented in Table 5. The initial BW of control, PF, PF+GL, and PF+GL+SBM were 738.6, 712.9, 729.1, and 718.2 kg, respectively, with no significant differences among treatments. The final BW of Control, PF, PF+GL, and PF+GL+SBM were 814.4, 803.6, 828.4, and 812.7 kg, respectively, with no significant differences among treatments.

Prior to the *in vivo* experiment, a palatability trial was conducted using the experimental TMR diets of control, PF, PF+GL, and PF+GL+SBM as a preliminary test. Eight early-fattening and five late-fattening Hanwoo steers were placed in four pens. The four experimental diets were fed twice a day for 1 h at 0900 and 1700, and the residuals were measured. The locations of the four diets were randomized at every 5 days for a total of 15 days, and animals were provided sufficient amounts of feeds to prevent consuming feeds from other treatments due to lack of feeds [19]. The mean DMI of control, PF, PF+GL, and PF+GL+SBM were 1.56, 2.90, 3.83, and 3.98 kg/h, respectively, indicating that the diets of PF, PF+GL, and PF+GL+SBM were more palatable than control (p<0.05). Consequently, added protected fat and glycerol can be assumed to have a positive effect on palatability for Hanwoo steer.

The DMI was not significantly different among all treatments during the whole experiment period, even though feeds were offered *ad libitum* for the late-fattening period. According to NRC (2016) [9], feed intake decreases when feeding diets with higher energy levels. In this study, the energy level was increased by 10% in PF and PF+GL, and both energy and protein levels were increased by 10% in PF+GL+ SBM compared to Control, but no decrease in DMI was observed. In the 1st period with relatively high THI, the DMI of control, PF, PF+GL, and PF+GL+SBM was measured at 7.00, 6.98, 7.01, and 6.94 kg, respectively. This indicates an approximately 33% decrease in DMI compared to the daily TMR feeding amount of 10.5 kg/d (DM basis; assuming an as-fed 15 kg/d including 30% moisture) recommended for late-fattening Hanwoo steers in NIAS (2017) [12]. In addition, compared to the 4th period, which entered the comfort stage after passing the high stress period, DMI in the 1st period decreased by about 30%. It is worth noting that the average THI of the 1st period was 87.0, which is a relatively low level of heat stress for a severe (THI 85 to 99) condition, but the decrease in DMI was very significant. The DMI in the 2nd period was 8.25, 7.80, 8.30, and 8.28 kg/d for control, PF, PF+GL, and PF+GL+SBM, respectively, which is approximately 18% less than the 4th period. Entering the 3rd period following the heat stress period in September, the DMI was 9.64, 9.32, 9.55, and 9.53 kg/d, and DMI in the 4th period was 9.94, 10.00, 10.01, and 10.01 kg/d, respectively. The decrease in DMI of ruminants under heat stress environments results in physiological changes due to excessive heat accumulation in the body [2,20]. Reduced DMI can cause serious economic losses through reduced productivity because the animals eating less would be unable to fulfill their nutrient requirements. In addition, ruminants have a physiologic response to high THI by increasing heat dissipation through increased peripheral blood volume and respiratory rate, resulting in an increase in metabolic rate as body temperature increases [6]. Under these circumstances, the energy requirements for maintenance may increase to 110% [6], and the use of energy feeds should be considered both to meet increased energy requirement and to compensate for the decrease in DMI [21].

In the 1st period, the ADG of control, PF, PF+GL, and PF+GL+SBM were 0.45, 0.74, 0.71, and 0.65 kg/d, respectively, showing higher values of treatment groups compared to control (p<0.05). In the 2nd period, the ADG of PF (0.85), PF+GL (0.86), and PF+GL+SBM (0.92) were higher than that of Control (0.67 kg/d) (p<0.05). Considering the 1st and 2nd period of the THI stages of fattening Hanwoo steers suggested by NIAS (2022) [6] were severe (85 to 99) and moderate (THI 82 to 84), respectively, ADG during these periods were significantly improved in all treatments fed 10% additional energy alone or energy and protein levels. In the 3rd period of post-hot season, ADG tended to improve only numerically in all treatments compared to control. On the other hand, there were no significant differences in ADG among treatments in the 4th period. The reason for this is unclear, but it is possible that the BW gain effect was not fully expressed during the late-fattening period just before transporting to a slaughterhouse. Furthermore, as the animal’s BW approaches 800 kg, the energy utilization rate gradually decreases, it is likely that the extra energy may not be expressed in BW [6]. The ADG of Control, PF, PF+GL, and PF+GL+SBM in the whole period (16 weeks) was 0.65, 0.79, 0.85, and 0.83 kg/d, respectively, which was higher in all treatments compared to Control primarily due to the high difference during the 1st and 2nd period (p<0.05).

According to NIAS (2017) [12], the EE contents in beef cattle diets should be 2% to 5% of the total DMI. In this experiment, the EE of the control diet was 5.35%, which was up to the limit (Table 2). However, in treatment groups that increased energy or energy and protein levels, EE contents in the diets exceeded 10%. Although 5% EE is generally recommended as an upper limit, additional feeding in the form of protected fat is acceptable because it does not affect rumen fermentation. It is also common practice in the dairy industry to feed up to 3% protected fat as a top dressing during the heat stress season [22].

Glycerol can partially replace corn and the inclusion of up to 15% in the diet of late-fattening steers does not affect productivity and carcass characteristics [23]. However, in this study, it was not possible to increase the energy level up to 10% by using glycerol alone when formulating a ratio based on NIAS (2017) [12]. No differences were observed in DMI and ADG due to the presence of glycerol addition, i.e. PF vs PF+GL. Meanwhile, when comparing the economic feasibility of glycerol with a protected fat, glycerol is not recommended as a good source of energy for the diet of heat stress.

When PF+GL+SBM increased protein levels by 10% was compared to PF+GL, there was no clear advantage in ADG of late-fattening Hanwoo steers. This may be a result of the additional energy required to convert excess ammonia to urea by increasing CP levels without considering rumen-protected protein [6]. On the other hand, Kim et al [15] conducted experiments on Hanwoo calves under mild (THI 71 to 74), moderate (THI 81 to 83), and severe (THI 89 to 91) conditions by fixing the energy level in the diet of 72% to 74% TDN and varying the CP level to 12.5%, 15.0%, and 17.5%. The results showed that feeding 17.5% CP under severe improved ADG by about 61%, compared to feeding 12.5%. However, Hanwoo steers in this study were at a stage of late fattening when energy level is relatively more important than CP for maintenance and marbling. Thus, additional feeding of soybean meal may not have a clear effect on productivity in this experiment.

The FCR of the 1st and 2nd period was improved in all treatments compared to control (p<0.05). The FCR during the 3rd and 4th period did not show significant differences among treatment groups. However, in the whole experimental period, FCR was significantly lower in all treatments (p< 0.05) compared to control (13.50). Similar to the ADG results, there was no significant difference between increased energy level treatments (PF and PF+GL) and PF+GL+SBM. Therefore, the results of this study suggest that feeding TMR diets with 110% increased energy level has a positive effect on mitigating productivity decline due to heat stress in late-fattening Hanwoo steers. However, the effect of increasing the protein level by 10% was not clear, so further experiments considering the level of rumen-protected protein will be necessary.

### Physiological parameters

**Figure 1:**
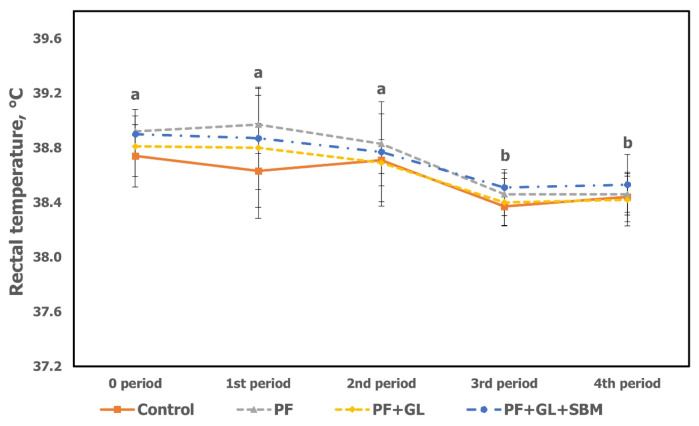
Changes in rectal temperature under heat stress of late-fattening Hanwoo steers during experimental period. Values are means±standard error for each treatment. ^a,b^ Mean values with different letters differ (p<0.05) by experimental periods, not by treatments.

The RT results of late-fattening Hanwoo steers fed diets with a 10% increase in energy and protein contents using protected fat, glycerol, and soybean meal under heat stress are shown in Table 6 and Figure 1. The RT is the most common method of measuring body temperature in livestock, and an increase in RT of ruminants indicates increased metabolic heat production in the body [24]. In this study, RT was not significantly different among treatments during the whole experimental period. However, when compared by periods, the mean RT was higher in the following order: 4th (10/28; 38.48°C), 3rd (9/30; 38.43°C), 2nd (9/2; 38.75°C), 1st (8/5; 38.81°C), and 0 period (7/9; 38.84°C) (p<0.05). Both the 0 and 1st period at Severe stage and the 2nd period at Moderate stage were higher values compared to the 3rd and 4th period of comfort (p<0.05; Figure 1). Thus, it was confirmed that the animals were already experiencing heat stress from the 0 period. The previous study [25] that measured the RT of early-fattening Hanwoo steers in chambers with precisely controlled AT and RH showed that it was 37.39°C, 37.80°C, 38.65°C, and 39.20°C in comfort (THI 73 to 75), mild (THI 77 to 79), moderate (THI 82 to 84), and severe (THI 85 to 86), respectively (p<0.05), and the difference between severe and comfort was approximately 1.8°C. The NIAS (2022) [6] reported that the RT of fattening steers was 39.3 for severe, 38.9 to 39.2 for moderate, and 38.5°C to 38.8°C for mild. The results from this study were lower than those of Woo et al [25] and NIAS (2022) [6]. The reason is probably that this study was conducted in an outdoor environment and the animals had time to recover from the heat stress by natural wind and at night when the THI was relatively lower. Although RT in ruminants is known to be positively correlated with THI (R^2^ = 0.62) [26], the significant decrease in the DMI of the 1st and 2nd period compared to the 3rd and 4th period indicated that this experiment was conducted under sufficient heat stress conditions.

**Figure 2:**
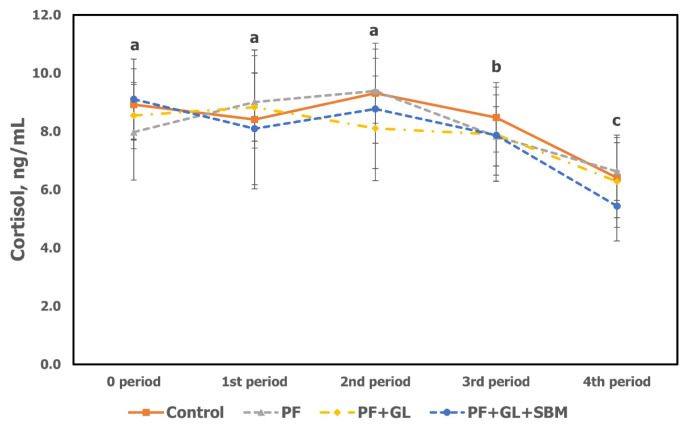
Changes in cortisol level under heat stress of late-fattening Hanwoo steers during experimental period. Values are means±standard error for each treatment. ^a,b^ Mean values with different letters differ (p<0.05) by experimental periods, not by treatments.

Serum cortisol in this study was not significant among treatments during the whole experimental period (Table 6; Figure 2). However, when comparing between periods, the mean serum cortisol concentrations were 8.63 (0 period), 8.59 (1st), 8.89 (2nd), 8.02 (3rd), and 6.19 ng/mL (4th). The serum cortisol concentrations in 0, 1st (severe), and 2nd period (moderate) were the highest, 3rd period was higher than 4th period at the comfort level (p<0.05; Figure 2). In the experiment using a climatic controlled chamber, serum cortisol levels in early-fattening Hanwoo steers were higher in the order of severe (17.17), moderate (15.57), mild (13.51), and comfort (11.98 ng/mL) phases [25]. In another study [27] on Hanwoo steers in an external environment, increased blood cortisol levels were observed after exposure to THI 80 to 87 (9.87) compared to THI 64 to 71 (1.91) and THI 72 to 79 (5.13 ng/mL). The concentration of cortisol, a hormone secreted from the adrenal cortex, increases under heat stress conditions [28]. There is a positive correlation (R^2^ = 0.59) between cortisol and THI [26]. Based on the results of this study and other studies, the NIAS (2022) [6] reported that blood cortisol levels of 1 to 4, 5 to 6, and 6 ng/mL or more in mild, moderate, and severe, respectively, but these values are considered too low. In addition, cortisol concentrations in the 0 and 1st period of this study were higher than those of the 3rd and 4th period, indicating that animals experienced high levels of heat stress during the 1st period.

The blood parameters of animals measured at the beginning of the experiment and every 4 weeks are presented in Table 6. Concentrations of serum glucose showed no significant differences among treatments. However, the glucose concentrations in 0 and 1st period were the lowest, and 3rd period was lower than 4th period (p<0.05). Serum glucose is the primary energy source utilized by ruminants during heat stress [29]. Glycerol is mainly converted to propionic acid by rumen microorganisms and enters the glucogenic pathway to temporarily increase blood glucose, which can be directly used as an energy source [30]. Thus, higher glucose level indicates that glycerol in PF+GL and PF+GL+SBM contributes to energy supply during heat stress. Protected fat may not have a direct effect on blood glucose levels because they do not interfere with rumen fermentation and can be absorbed in the small intestine to provide more energy for ruminants [31]. When used in combination with glycerol, protected fat can be used for energy storage while glycerol may act as an energy source. Several studies [7,26] have reported a decrease in blood glucose due to heat stress. Firstly, this is because energy requirements for homeostasis control increase due to an increase in body temperature under heat stress, while energy intake decreases, and blood glucose is rapidly consumed. Secondly, the endocrine system may be affected under heat stress conditions, having a negative effect on the process of gluconeogenesis [32]. In this study, the serum glucose concentration of late-fattening Hanwoo steers tended to decrease in the 0, 1st, and 2nd period of heat stress compared to the 3rd and 4th period of following heat stress.

Concentrations of blood TG and TCHO were not significantly different among treatments during the entire experiment. The TG concentration showed an interaction of treatment and time (p<0.05) with a relatively low treatment effect. The TCHO concentration in 0 period at Severe was higher than that of other periods (p<0.05). Another study reported that the TCHO and TG concentrations tend to increase with higher energy intake and better growth performance in ruminants [33]. Kang et al [7] reported that supplemental feeding of protected fat to Hanwoo steers did not affect blood TG and TCHO concentrations compared to control. Therefore, the relationship between increased energy and protein levels and blood TG and TCHO concentrations during heat stress is considered unclear.

Blood NEFA showed no significant differences among treatments during whole period. The NEFA concentration was a low time effect, suggesting an interaction between treatment and time. This is due to the fact that blood NEFA increases by breaking down the fat stored in the body and releasing it into the blood to supplement the required energy because the energy intake is insufficient due to heat stress [34]. In contrast, treatments that increased energy and (or) protein levels appear to have lower blood NEFA levels because sufficient energy and protein were supplied.

Blood BUN concentrations showed no significant differences among treatments in the whole period. However, the mean BUN concentrations of 0, 1st, and 2nd period were higher than those of 3rd and 4th period (p<0.05). The mechanisms of the increase in BUN were unclear, but first, it may be attributed to the increased production of rumen ammonia or proteolysis of skeletal muscle [35]. Second, heat-stressed ruminants have increased BUN levels compared to thermoneutral control, suggesting that rumen ammonia was inefficiently synthesized into microbial CP [35]. Thus, it was possible that blood ammonia concentrations were increased by surplus rumen ammonia [36]. Overall, blood parameters that can determine nutritional levels, such as glucose, TG, TCHO, NEFA, and BUN, are not appropriate indicators for determining the degree of heat stress.

### Carcass characteristics

Carcass performance for supplemental feeding of glycerol, protective fat, and soybean meal to late-fattening Hanwoo steers during the hot and post-hot seasons is presented in Table 7. Carcass yield parameters including carcass weight, *longissimus* muscle area, backfat thickness, meat index and carcass grade score were not affected by the increased energy and protein levels. Carcass grade items such as marbling score, meat color, fat color, texture, maturity, and meat grade score were also not significantly different among all treatments.

Research comparing carcass performance of beef cattle feeding protected fat or glycerol is very limited. Piao et al [37] found that supplemental feeding of approximately 3% glycerol to late-fattening Hanwoo steers with the same TDN content resulted in no significant differences in carcass yield and quality grades. In an experiment by Lee et al [38], carcass weights of steers fed rumen-protected oleic acid for 90 days were about 8% heavier than the control group, but the carcass index was slightly lower than the control. In addition, backfat, marbling score, and loin area tended to be higher in the oleic acid-fed treatment than in the control group. Although there was a high correlation between live weight and carcass weight percentage [39], feeding higher energy diets does not necessarily increase carcass leanness [40]. Our results showed that additional energy and protein levels over 4 months did not affect carcass characteristics, but led to improvements in ADG.

### Animal behaviors

Changes in animal behaviors between hot season (1st period) and post-hot season (3rd period) are presented in Table 8. Animal behaviors were not different among treatment groups within the same period. The purpose of measuring animal behaviors in this experiment was not to focus on changes caused by diet effects, but to examine changes while experiencing heat stress. Accordingly, the significance of behavioral changes by period in hot season and post-hot season was verified.

Regardless of treatments, lying decreased approximately 43% in hot season of 1st period compared to post-hot season of 3rd period, whereas total standing increased 48% (p<0.05). There was also a 62% decrease in walking (p<0.05). Kim et al [27] reported that the lying of early-fattening period of Hanwoo steers in outdoor environment decreased by 25% from severe (THI 80 to 87) compared to comfort (THI 64 to 71), while standing increased by 11% (p<0.05). It is known that the increase in standing time is intended to effectively reduce body heat by minimizing the area in contact with the ground and expanding the body surface area exposed to the air [9,41].

Eating decreased 38% in hot season compared to post-hot season (p<0.05). This is because DMI decreased by 36% due to heat stress during the 1st period compared to the 3rd period. On the other hand, drinking increased by 54% in hot season compared to post-hot season (p<0.05). The increase in drinking time could be attributed to the increased consumption of water to lower body temperature under heat stress [6,25].

Rumination during standing was 53% higher in hot season than in post-hot season, while rumination during lying was about 33% lower (p<0.05). This is because the relative times of rumination during standing and rumination during lying changed, considering that the total standing time increased and lying decreased under heat stress. In addition, the total rumination time was relatively lower in hot season than in post-hot season, which may be associated with the decrease in feed intake [20]. Therefore, these results meaningfully show how the behaviors of ruminants change in a heat stress condition.

## CONCLUSION

This study investigated the dietary effects of protected fat, glycerol, and soybean meal on productivity, physiological parameters, carcass characteristics, and behavioral measurements of late-fattening Hanwoo steers under heat stress conditions. There was no difference in DMI among treatments., but ADG and FCR were higher in treatment groups (PF, PF+GL, PF+GL+SBM) compared to control during 1st (severe) and 2nd (moderate) period. There were no treatment effects for RT, serum cortisol, and behaviors during the whole period. Both RT and serum cortisol level were elevated with increased THI. Blood parameters lacked reliability for detecting heat stress. Carcass yield and grade were unaffected by increased TDN and CP. Hot season (1st period) induced behavioral changes (less lying, more standing, less walking, reduced eating) with a notable 54% increase in drinking compared to post-hot season (3rd period). Overall, feeding of TMR diets increasing TDN level by 10% under heat stress conditions has a positive effect on preventing productivity reduction in late-fattening Hanwoo steers without compromising palatability.

## Figures and Tables

**Table 1:** Formula of experimental total mixed ration diets for Hanwoo steers of late-fattening period

Items, % (as-fed)	Treatments^1)^			

	Control	PF	PF+GL	PF+GL+SBM
Glycerol	-	-	5.00	5.00
Protected fat	-	3.32	3.02	3.03
Soybean meal	-	1.82	4.41	7.56
Wheat bran	18.00	16.45	18.04	19.28
Corn flake	32.20	30.34	23.78	20.80
Perilla meal	8.40	7.68	6.02	5.26
Barley brewers grains	20.00	18.28	14.33	12.53
Rice straw	11.70	11.69	11.69	11.69
Water	6.50	8.22	11.52	12.63
Vitamin and mineral premix	0.24	0.24	0.24	0.24
Limestone	1.18	1.18	1.18	1.18
Salt	0.39	0.39	0.39	0.39
Sodium bicarbonate	0.39	0.39	0.39	0.39
Total	100	100	100	100

1) Control, no supplements: PF, 10% more energy with protected fat; PF+GL, 10% more energy with protected fat and glycerol; PF+GL+SBM, 10% more energy and protein with protected fat, glycerol, and soybean meal.

**Table 2:** Chemical composition of experimental total mixed ration diets

Items, % (DM basis)	Treatments^1)^			

	Control	PF	PF+GL	PF+GL+SBM
DM	69.21	67.20	67.96	65.86
CP	14.99	15.03	15.01	16.52
EE	5.35	10.54	10.58	10.46
Ash	8.02	5.75	8.37	8.12
CF	9.87	9.07	8.34	7.99
NDF	36.76	33.26	29.19	31.77
ADF	13.68	12.25	13.15	11.32
ADL	3.24	2.70	2.37	2.07
TDN	76.04	83.66	83.67	83.66

DM, dry matter; CP, crude protein; EE, ether extract; CF, crude fiber; NDF, neutral detergent-insoluble fiber; ADF, acid detergent-insoluble fiber; ADL, acid detergent lignin; TDN, total digestible nutrients.

1) Control, no supplements: PF, 10% more energy with protected fat; PF+GL, 10% more energy with protected fat and glycerol; PF+GL+SBM, 10% more energy and protein with protected fat, glycerol, and soybean meal.

**Table 3:** Mean, maximum, and minimum values of ambient temperature, relative humidity, and THI during the experimental period

Items	Experimental period^1)^				SEM^2)^	p-value

	1st	2nd	3rd	4th		
Ambient temperature (°C)						
Mean	32.8^a^	27.5^b^	25.1^c^	21.3^d^	0.52	<0.001
Maximum	45.1^a^	33.6^b^	32.2^bc^	31.9^c^	1.01	<0.001
Minimum	27.7^a^	19.7^b^	17.6^c^	9.4^d^	0.76	<0.001
Relative humidity (%)						
Mean	74.1^b^	76.5^a^	72.3^c^	56.5^d^	2.21	<0.001
Maximum	92.4^a^	92.0^b^	91.8^b^	87.2^c^	0.82	<0.001
Minimum	31.7^d^	50.3^a^	38.3^b^	31.8^c^	2.84	<0.001
THI^3)^						
Mean	87.0^a^	82.8^b^	71.4^c^	68.1^d^	0.57	<0.001
Maximum	98.2^a^	86.6^d^	88.5^b^	87.4^c^	0.68	<0.001
Minimum	80.5^a^	64.6^b^	59.3^c^	53.5^d^	0.84	<0.001

1) 1st period (0 to 4 wk), 210709–210805; 2nd period (5 to 8 wk), 210806–210902; 3rd period (9 to 12 wk), 210903–210930; 4th period (13 to 16 wk), 211001–211028.

2) SEM, standard error of the means.

3) THI, temperature-humidity index; (1.8 × ambient temperature + 32) – (0.55 – 0.0055 × relative humidity) × (1.8 × ambient temperature – 26) [16].

a–d Means within a row without a common superscript letter differ (p<0.05).

**Table 4:** Behaviors and definitions of Hanwoo steers recorded by CCTV

Item	Definition
Lying	Legs and lower abdomen contact with the ground
Total standing	All legs are extended and supporting the body
Walking	Moving over three footprints in a standing position
Eating	Standing and bowing down to the feed
Drinking	Head over or in the water trough
Rumination during lying	Regurgitating and chewing during lying without feed
Rumination during standing	Regurgitating and chewing during standing without feed

**Table 5:** Dietary effects of protected fat, glycerol, and soybean meal on growth performance of Hanwoo steers in late-fattening period under heat stress conditions

Items	Treatments^1)^				SEM	p-value

	Control	PF	PF+GL	PF+GL+SBM		
BW (kg)						
0 wk	738.6	712.9	729.1	718.2	20.013	0.801
4th wk	751.1	733.8	748.8	736.5	22.307	0.832
8th wk	770.0	756.6	775.7	762.7	22.329	0.910
12th wk	793.6	784.2	804.9	787.0	22.508	0.912
16th wk	814.4	803.6	828.4	812.7	22.572	0.890
DMI^2)^ (kg/d)						
1st period	7.00	6.98	7.01	6.94	0.030	0.335
2nd period	8.25	7.80	8.30	8.28	0.490	0.477
3rd period	9.64	9.32	9.55	9.53	0.243	0.346
4th period	9.94	10.00	10.01	10.01	0.069	0.503
Whole period	8.84	8.75	8.85	8.78	0.111	0.589
ADG^2)^ (kg/d)						
1st period	0.45^b^	0.74^a^	0.71^a^	0.65^a^	0.063	0.009
2nd period	0.67^b^	0.85^a^	0.86^a^	0.92^a^	0.043	0.001
3rd period	0.81	0.98	0.96	0.87	0.081	0.237
4th period	0.73	0.70	0.81	0.79	0.085	0.875
Whole period	0.68^b^	0.82^a^	0.85^a^	0.83^a^	0.040	0.002
FCR^2)^ (F/G)						
1st period	15.56^a^	9.44^b^	9.87^b^	10.69^b^	2.040	0.007
2nd period	12.31^a^	9.18^b^	9.65^b^	9.00^b^	1.852	0.003
3rd period	11.90	9.52	9.95	10.95	1.249	0.176
4th period	13.63	14.28	12.36	12.67	1.653	0.176
Whole period	13.50^a^	10.66^b^	10.88^b^	11.39^b^	1.098	0.027

SEM, standard error of the means; BW, body weight; DMI, dry matter intake; ADG, average daily gain; FCR, feed conversion ratio.

1) Control, no supplements; PF, 10% more energy with protected fat; PF+GL, 10% more energy with protected fat and glycerol; PF+GL+SBM, 10% more energy and protein with protected fat, glycerol, and soybean meal.

2) 1st period (0 to 4 wk), 210709–210805; 2nd period (5 to 8 wk), 210806–210902; 3rd period (9 to 12 wk), 210903–210930; 4th period (13 to 16 wk), 211001–211028; Whole period (0 to 16 wk), 210709–211028.

a,b Means within a row without a common superscript letter differ (p<0.05).

**Table 6:** Dietary effects of protected fat, glycerol, and soybean meal on physiological parameters of Hanwoo steers in late-fattening period under heat stress conditions

Period^1)^	Treatments^2)^				SEM^3)^	p-values		
	
	Control	PF	PF+GL	PF+GL+SBM		Diet	Day	Diet×day
	----------------------------------- Rectal temperature (°C) --------------------------							
Mean (Diet)	38.58	38.74	38.62	38.72	0.069	0.387	<0.001	0.994
0	38.74	38.92	38.81	38.90				
1st	38.63	38.97	38.80	38.87				
2nd	38.71	38.83	38.69	38.77				
3rd	38.37	38.46	38.40	38.51				
4th	38.44	38.46	38.42	38.53				
	-------------------------------------- Cortisol (ng/mL) -------------------------------------							
Mean (Diet)	8.31	8.17	7.93	7.85	0.241	0.535	<0.001	0.755
0	8.92	7.97	8.54	9.10				
1st	8.41	9.01	8.83	8.09				
2nd	9.31	9.39	8.10	8.77				
3rd	8.48	7.83	7.90	7.87				
4th	6.41	6.62	6.28	5.43				
	-------------------------------------- Glucose (mg/dL) -------------------------------------							
Mean (Diet)	74.45	76.54	76.25	76.28	0.550	0.086	<0.001	0.679
0	71.63	73.28	74.94	73.64				
1st	72.19	75.24	75.29	75.73				
2nd	74.52	77.36	74.20	76.15				
3rd	75.32	77.48	77.48	76.12				
4th	78.60	79.32	80.38	79.78				
	----------------------------------- Triglyceride (mg/dL) -----------------------------------							
Mean (Diet)	16.80	16.51	17.06	18.37	0.478	0.097	0.485	0.025
0	16.08	15.76	17.76	19.23				
1st	15.83	15.74	19.59	19.93				
2nd	17.18	16.37	15.62	17.16				
3rd	17.58	17.71	14.93	18.80				
4th	17.32	17.97	17.39	16.73				
	------------------------------- Total cholesterol (mg/dL) --------------------------------							
Mean (Diet)	165.5	179.8	171.4	172.7	4.940	0.330	0.003	0.946
0	189.4	197.1	192.2	181.6				
1st	167.9	169.2	163.9	177.2				
2nd	155.2	166.9	157.3	164.2				
3rd	150.3	172.7	171.7	174.4				
4th	164.4	192.9	172.0	165.9				
	----------------------------- Non-esterified fatty acid (μEq/L) ------------------------							
Mean (Diet)	216.2	197.2	199.0	200.7	8.249	0.391	0.076	0.006
0	202.3	213.4	206.9	216.2				
1st	247.1	225.0	207.1	176.3				
2nd	178.0	185.8	197.2	228.6				
3rd	226.7	175.0	184.0	164.4				
4th	226.8	187.0	199.9	217.9				
	---------------------------- Blood urea nitrogen (mg/dL) -----------------------------							
Mean (Diet)	15.71	15.52	15.62	15.10	0.394	0.696	<0.001	<0.001
0	14.33	14.47	14.69	14.81				
1st	12.82	14.87	14.38	16.76				
2nd	15.26	12.79	16.92	14.10				
3rd	17.83	16.72	14.89	16.07				
4th	18.32	18.77	17.23	13.75				

1) 0 period, measured on Jul 9; 1st period, Aug 5; 2nd period, Sep 2; 3rd period, Sep 30; 4th period, Oct 28.

2) Control, no supplements; PF, 10% more energy with protected fat; PF+GL, 10% more energy with protected fat and glycerol; PF+GL+SBM, 10% more energy and protein with protected fat, glycerol, and soybean meal.

3) SEM, standard error of the means.

**Table 7:** Carcass weight and characteristics of late-fattening Hanwoo steers fed supplemental protected fat, glycerol, and soybean meal

Items	Treatments^1)^				SEM^2)^	p-value

	Control	PF	PF+GL	PF+GL+SBM		
Yield						
Carcass weight (kg)	472.4	486.2	450.6	489.1	14.71	0.210
Longissimus muscle area^3)^ (cm^2^)	93.56	98.78	97.78	102.22	3.201	0.310
Backfat thickness (mm)	15.00	17.56	14.33	15.67	1.955	0.517
Index^4)^	60.77	60.30	61.43	60.99	0.655	0.446
Grade score^5)^	1.67	1.89	1.78	1.67	0.279	0.932
Grade ratio^6)^, A:B:C (%)	0:78:22	33:11:56	33:33:33	11:56:33	-	-
Quality						
Marbling score^7)^	6.78	6.22	6.33	7.11	0.583	0.688
Meat color^8)^	4.89	4.56	4.67	4.67	0.192	0.429
Fat color^9)^	3.22	3.22	3.00	3.00	0.105	0.225
Texture^10)^	1.44	1.33	1.11	1.22	0.152	0.457
Maturity^11)^	2.11	2.67	2.33	2.33	0.165	0.098
Grade score^12)^	2.22	2.33	2.00	2.22	0.301	0.800
Grade ratio^13)^ 1++:1+:1 (%)	44:44:11	33:33:33	56:11:33	44:44:11	-	-

n = 9 steers per treatment.

1) Control, no supplements: PF, 10% more energy with protected fat; PF+GL, 10% more energy with protected fat and glycerol; PF+GL+SBM, 10% more energy and protein with protected fat, glycerol, and soybean meal.

2) SEM, standard error of the means.

3) Measured from longissimus muscle at 13th rib.

4) Calculated using the following equation: 68.184–[0.625×backfat thickness (mm)]+[0.130 longissimus muscle area (cm^2^)]–[0.024×carcass weight (kg)]; A grade, >67.20; B grade, 63.30–67.20; C grade, <63.30.

5) Yield grade: A, 1; B, 2; C, 3.

6) A, high yield; C low yield.

7) 1, devoid; 9, very abundant.

8) 1, bright red; 7, dark red.

9) 1, creamy white; 7, yellowish.

10) 1, soft; 3, firm.

11) 1, youthful; 9, mature.

12) Quality grade: 1^++^ = 1; 1^+^ = 2; 1 = 3 points; Average value calculated by assigning points to each meat quality grade.

13) 1^++^ = very high quality; 1^+^ = high quality; 1 = good quality; 2 = low quality; 3 = very low quality.

**Table 8:** Observation of animal behaviors (min/d) of late-fattening Hanwoo steers fed supplemental protected fat, glycerol, and soybean meal under heat stress conditions

Items^1)^	Period^2)^		SEM^3)^	p-value

	1st	3rd		
Lying				
Control	366.8^b^	702.9^a^	33.86	0.020
PF	409.9^b^	724.4^a^	11.29	0.003
PF+GL	443.3^b^	677.6^a^	7.75	0.015
PF+GL+SBM	391.6^b^	738.9^a^	47.09	0.013
Total standing^4)^				
Control	728.8^a^	388.3^b^	6.71	0.001
PF	659.9^a^	333.7^b^	44.97	0.023
PF+GL	736.5^a^	371.3^b^	41.40	0.025
PF+GL+SBM	720.1^a^	398.3^b^	20.10	0.047
Walking				
Control	13.1^b^	36.9^a^	0.95	0.003
PF	10.7^b^	31.9^a^	1.39	0.007
PF+GL	18.6^b^	37.0^a^	1.41	0.013
PF+GL+SBM	13.5^b^	40.2^a^	3.68	0.031
Eating				
Control	161.4^b^	268.7^a^	12.25	0.025
PF	140.5^b^	220.0^a^	7.01	0.015
PF+GL	147.4^b^	228.0^a^	10.32	0.031
PF+GL+SBM	139.9^b^	237.0^a^	14.12	0.040
Drinking				
Control	23.4^a^	10.8^b^	1.82	0.040
PF	29.7^a^	12.8^b^	1.12	0.009
PF+GL	25.4^a^	13.0^b^	1.40	0.024
PF+GL+SBM	22.1^a^	9.9^b^	0.80	0.037
Rumination/standing				
Control	178.2^a^	98.5^b^	5.45	0.030
PF	184.2^a^	76.0^b^	9.69	0.031
PF+GL	166.1^a^	89.3^b^	7.42	0.018
PF+GL+SBM	182.3^a^	67.2^b^	9.87	0.028
Rumination/lying				
Control	313.2^b^	526.8^a^	12.18	0.020
PF	302.0^b^	511.8^a^	28.52	0.027
PF+GL	389.0^b^	504.1^a^	19.09	0.034
PF+GL+SBM	297.0^b^	389.5^a^	12.92	0.037

1) Control, no supplements: PF, 10% more energy with protected fat; PF+GL, 10% more energy with protected fat and glycerol; PF+GL+SBM, 10% more energy and protein with protected fat, glycerol, and soybean meal.

2) 1st period, Severe; 3rd period, Comfort; observed when the same temperature-humidity index level lasted more than 5 days, respectively.

3) SEM, standard error of the means.

4) Include eating, rumination, walking and drinking.

a,b Means within a row in a same parameter that do not share a letter differ (p<0.05).
